# Retraction: Comparative assessment of various supplementary diets on commercial honey bee (*Apis mellifera*) health and colony performance

**DOI:** 10.1371/journal.pone.0275146

**Published:** 2022-09-30

**Authors:** The PLOS ONE Editors

The *PLOS ONE* Editors retract this article [1] because it was identified as one of a series of submissions for which we have concerns about authorship, competing interests, and peer review. We regret that the issues were not addressed prior to the article’s publication.

SA responded but expressed neither agreement nor disagreement with the editorial decision. KAK, SAK, HAG, and AG either did not respond directly or could not be reached.
